# Efficacy and Safety of Selexipag Treatment in Connective Tissue Disease-Associated Pulmonary Arterial Hypertension with Concomitant Interstitial Lung Disease

**DOI:** 10.3390/life15060974

**Published:** 2025-06-18

**Authors:** Chebly Dagher, Maria Akiki, Kristen Swanson, Brett Carollo, Harrison W. Farber, Raj Parikh

**Affiliations:** 1 Department of Internal Medicine, University of Connecticut, Farmington, CT 06030, USA; 2 Division of Pulmonary, Critical Care and Sleep, Hartford Hospital, Hartford, CT 06102, USA; 3 Division of Pulmonary, Sleep and Critical Care Medicine, Tufts Medical Center, Boston, MA 02111, USA

**Keywords:** pulmonary hypertension, selexipag, interstitial lung disease, connective tissue disease, ventilation–perfusion mismatch

## Abstract

Patients with connective tissue disease-associated pulmonary arterial hypertension (CTD-PAH) and concomitant interstitial lung disease (ILD) are particularly challenging to manage due to concerns about ventilation–perfusion mismatch with systemic vasodilators. In this case series, we evaluated the effects of selexipag in eight prostacyclin-naïve CTD-PAH patients with concomitant ILD. Clinical, functional, and laboratory data were collected at baseline and after 16 weeks of treatment. After 16 weeks of treatment, the mean six-minute walk distance increased by 101.75 m (*p* < 0.05), and the mean estimated right ventricular systolic pressure decreased significantly (*p* < 0.05). Mean N-terminal pro b-type natriuretic peptide levels declined by 63%, though this reduction did not reach statistical significance. Importantly, supplemental oxygen requirements trended downward (*p* < 0.05) and pulmonary function tests remained stable. Pulmonary vasodilators have long been unsuccessfully studied in PH-ILD patients until the INCREASE trial. While other systemic agents used in PAH have not shown as much success as inhaled treprostinil in treating PH-ILD, our case series highlights the potential role of selexipag in patients with concomitant CTD-PAH and ILD. Further investigation of selexipag in pure Group 3 PH-ILD patients is warranted.

## 1. Introduction

Pulmonary hypertension (PH) is a complex condition characterized by elevated pressures within the pulmonary circulation, which can result from various underlying mechanisms. Based on the World Health Organization (WHO) categorizations, PH is classified into five groups: Group 1 pulmonary arterial hypertension (PAH), Group 2 PH due to left heart disease (PH-LHD), Group 3 PH caused by lung disease and/or hypoxia (PH-LD), Group 4 chronic thromboembolic PH (CTEPH), and Group 5 PH with unclear or multifactorial mechanisms [1,2]. Connective tissue disease (CTD) is the most common systemic disease associated with Group 1 PAH [3]. Meanwhile, Group 3 PH can occur as a result of various interstitial lung diseases (ILD), including idiopathic pulmonary fibrosis (IPF), hypersensitivity pneumonitis (HP), and nonspecific interstitial pneumonia (NSIP); CTD is also a common risk factor for ILD, independent of PH. In these ILD patients, the development of concomitant pulmonary vascular disease is associated with increased rates of morbidity and mortality [4,5].

Group 3 PH has a different pathophysiology from Group 1 PAH, often making PAH-specific treatments ineffective in this entity. This ineffectiveness arises because these PAH treatments dilate all pulmonary vessels, including those that do not participate in oxygen exchange due to reduced alveolar ventilation. Consequently, therapies tailored for PAH can theoretically cause a mismatch between ventilation and perfusion in PH-ILD patients, further impairing oxygenation and reducing exercise capacity [6]. The majority of randomized controlled trials evaluating PAH-specific treatments for PH-LD have resulted in treatment failures or even more detrimental outcomes in the treated cohort [2,7]. Consequently, CTD-PAH patients with concomitant ILD can pose significant management challenges when treating the underlying pulmonary vascular disease.

Selexipag is an oral pulmonary vasodilator that acts as a selective agonist for the prostacyclin receptor and has a different structure from prostacyclin [8]. It is approved for the treatment of PAH [4,9]; however, its efficacy in CTD-PAH patients with coexisting ILD remains uncertain. Its gradual titration strategy might reduce the occurrence of early treatment discontinuation in PH-ILD patients due to rapid ventilation–perfusion mismatch that is often associated with other pulmonary vasodilators [10]. As such, this case series evaluated the effects of selexipag in a cohort of CTD-PAH patients with concomitant CTD-ILD.

## 2. Materials and Methods

We retrospectively analyzed 8 CTD-PAH patients with concomitant ILD. CTD-PAH was defined as patients with known, underlying CTD and right heart catheterization (RHC) criteria for pre-capillary PH: mean pulmonary artery pressure (mPAP) > 20 mmHg, pulmonary capillary wedge pressure (PCWP) < 15 mmHg, and pulmonary vascular resistance >2 wood units (WU) [11]. The diagnosis of ILD was confirmed by the presence of diffuse parenchymal lung disease on chest CT imaging. Patients who were already receiving a prostacyclin agent for the treatment of PH were excluded. The study was approved by the Hartford Hospital Institutional Review Board (HH-25-48).

Clinical and demographic data were collected, including age, gender, type and duration of CTD, type of ILD, treatment for both PAH and ILD, diffusing capacity of the lungs for carbon monoxide (DLCO), and invasive hemodynamics from RHC. Additionally, baseline and follow-up data were collected for the following parameters: six-minute walk distance (6MWD in meters), N-terminal prohormone brain natriuretic peptide (NT-proBNP in pg/mL), echocardiographic estimation of right ventricular systolic pressure (eRVSP in mmHg), supplemental oxygen therapy (in liters), predicted and absolute forced expiratory volume in one second (FEV_1_, as a percentage and in liters, respectively), and predicted and absolute forced vital capacity (FVC, as a percentage and in liters, respectively). Data on treatment adjustments and hospitalizations were collected from the patients’ electronic medical records. Decisions regarding oxygen therapy were based on symptoms and an SpO_2_ level < 88% at rest and with exertion, per standard clinical practice.

A follow-up period of 16 weeks was selected for its established relevance and precedence in PH research. Notably, the INCREASE study, the largest to date in PH-ILD, demonstrated significant results at 16 weeks, which led to FDA approval of inhaled treprostinil for this indication. Furthermore, 16 weeks is a widely utilized evaluation period in PH trials, providing sufficient time to assess treatment efficacy and safety meaningfully [9,12,13]. A one-sample *t*-test was used to assess the significance of mean changes in clinical and functional outcomes after 16 weeks of selexipag treatment (α = 0.05; n = 8), with analyses performed in Python 3.11 as this approach is appropriate for evaluating within-group changes in the absence of a comparator arm.

## 3. Results

The median age of the eight patients was 51.5 years, and five were female. Five patients had mixed connective tissue disease (MCTD), while the remaining three had scleroderma. The median duration of CTD at the time of selexipag initiation was 2.5 years. All patients were receiving background therapy for PAH, consisting of a phosphodiesterase-5 inhibitor (PDE-5-I) and an endothelin receptor antagonist (ERA). None had previously received prostacyclin therapy. All patients had ILD confirmed by chest CT, with a median DLCO of 38.5% predicted (range: 28–50%). Six patients were classified as having fibrotic nonspecific interstitial pneumonia (NSIP), and two had non-fibrotic NSIP, based on HRCT findings reviewed by thoracic radiologists. Classification was confirmed through multidisciplinary discussion. ILD treatment included mycophenolate mofetil (5/8), hydroxychloroquine (7/8), and low-dose prednisone (1/8). At the time of PAH diagnosis, the average mPAP was 47 mmHg and the mean pulmonary vascular resistance (PVR) was 8.6 WU (Table 1 and Table 2).

All eight patients achieved a maintenance dose of selexipag 1600 mcg twice daily, after initiating at the starting dose of 200 mcg twice daily. The dose was titrated weekly or at a slower pace, depending on individual tolerance, and was dictated by specialty pharmacy nursing. The titration process was completed over 8 weeks, allowing patients to reach the target maintenance dose of 1600 mcg. Throughout the titration period, all patients experienced some side effects, primarily headaches and nausea. None of the patients discontinued selexipag therapy during the 16-week period.

**Figure 1:**
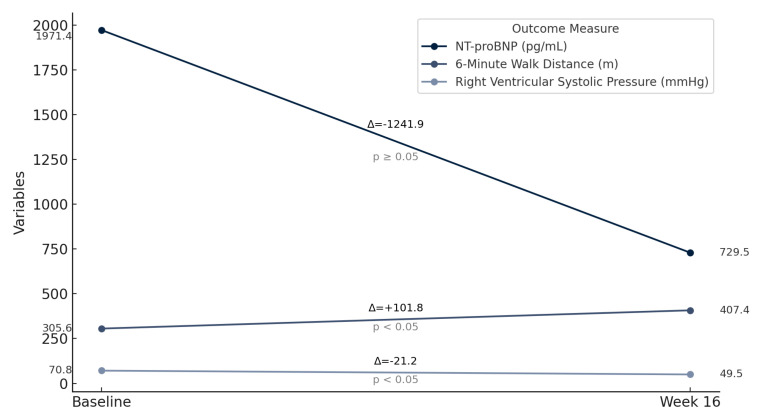
Improvement in 6-minute walk distance, NT-proBNP, and right ventricular systolic pressure post selexipag treatment.

At 16 weeks, the mean improvement in 6MWD was 101.8 m (t = −6.66, *p* < 0.05), corresponding to an average increase of 58%. NT-proBNP levels decreased in all eight patients, but this reduction was not statistically significant for the group as a whole (t = 1.86, *p* = 0.1048). Patient 4 showed the most notable reduction of 78.7%, from 4203 pg/mL to 937 pg/mL. Echocardiographic assessments of eRVSP revealed consistent reductions post-treatment, with values decreasing from the range of 55–90 mmHg at baseline to 41–67 mmHg at 16 weeks (t = 7.98, *p* < 0.05). Supplemental oxygen requirements decreased significantly in most patients (t = 2.65, *p* < 0.05): patients 1, 3, and 8 no longer needed oxygen, and the oxygen needs in patient 4 decreased from 4 L/min to 2 L/min. Absolute FVC decreased in patients 2, 5, and 7; however, this finding was not statistically significant (t = 1.39, *p* = 0.208). The mean predicted FVC declined from 60.5% at baseline to 58.9% at 16 weeks, representing a −1.63% change, which was also not statistically significant (t = 0.956, *p* = 0.371). Absolute FEV_1_ increased from 2.00 L at baseline to 2.06 L at 16 weeks (mean change: +0.063 L), but this change did not reach statistical significance (t = −1.93, *p* = 0.095). Predicted FEV_1_ rose slightly from 60.5% to 61.0% (mean change: +0.5%; t = 1.17, *p* = 0.280), again without statistical significance (Table 3 and Table 4; Figure 1).

## 4. Discussion

Our analysis of this cohort suggests that treatment with selexipag can improve exercise capacity, reduce eRVSP, and decrease oxygen requirements without precipitating ventilation–perfusion mismatch in CTD-PAH patients with concomitant parenchymal lung disease.

While treatment options for Group 1 PAH are well established, therapeutic strategies for PH-ILD are limited to inhaled treprostinil [14]. PH associated with ILD significantly contributes to morbidity and mortality in affected patients, underscoring an urgent need for targeted interventions [15,16]. Other pulmonary vasodilators that are not delivered via the inhaled route, have not shown benefits in PH-ILD. For example, the RISE-IIP study found that riociguat increased serious adverse events and mortality without improvement in exercise capacity (6MWD) in patients with Group 3 PH [6]. In addition, several endothelin receptor antagonists (ERA) have not shown benefits in the PH-ILD population [17,18,19,20,21,22]. While these findings raise concerns about the potential harm of using systemic pulmonary vasodilators in the context of ILD and PH, a preliminary study involving eight patients with PH-ILD demonstrated that selexipag effectively improved the 6MWD in half of the participants [23]. Notably, higher body mass index (BMI) and lower ratio of arterial oxygen partial pressure to fraction of inspired oxygen (PaO_2_/FiO_2_) levels were identified as potential indicators of a positive response, although no significant changes in hemodynamic parameters were observed [23].

The theoretical challenges of utilizing systemic pulmonary vasodilator agents in patients with PH-associated parenchymal lung disease center on concerns about worsening oxygenation due to increased ventilation–perfusion mismatch [16]. This explains the rationale and success of inhaled treprostinil observed in the INCREASE study; in that trial, inhaled treprostinil significantly improved exercise capacity, reduced NT-proBNP levels, lowered the risk of clinical worsening, and decreased lung disease exacerbations during the 16-week study, also, of importance, there was no observed decline in lung function [14]. The authors attributed the absence of worsened ventilation–perfusion mismatch to the inhaled delivery of treprostinil [14]. However, concern about ventilation–perfusion mismatch with systemic pulmonary vasodilators for PH-associated parenchymal lung disease may be offset by a corresponding increase in cardiac output, leading to improved tissue oxygen delivery [16]. Our eight CTD-PAH patients with concomitant ILD demonstrated that treatment with selexipag led to meaningful improvements in 6MWD and echocardiographic measures of hemodynamics, without evidence of worsening oxygenation. Importantly, both absolute and predicted FVC and FEV_1_ remained stable over the 16-week treatment period, with no statistically significant decline, further supporting the safety of selexipag in patients with PAH despite concomitant ILD. Some variability in pulmonary function was observed, which is expected in this population given the underlying progressive nature of ILD and the heterogeneity of the cohort. It is difficult to draw definitive conclusions about the factors influencing individual responses, though disease severity, comorbidities, and timing of treatment initiation may have contributed. While several oral PAH therapies have been studied in the treatment of PH-ILD, selexipag has yet to be evaluated in a large-scale trial.

A strength of this cohort is its real-world use of selexipag in clinical practice for patients with CTD-PAH and concurrent ILD. However, the study does have several limitations. The small sample size limits the findings; the results should be interpreted in this context. Additionally, the follow-up period of 16 weeks may not reflect the long-term efficacy and safety of selexipag in patients with PAH-CTD and concomitant ILD. Moreover, as a single-center study, the findings may be influenced by local practices and may not be broadly applicable to other settings. Some variables, such as NT-proBNP levels, did not reach statistical significance, likely due to the small sample size, highlighting the need for larger, long-term trials to validate these findings. Furthermore, multiple outcomes were assessed without formal correction for multiple comparisons, which may increase the risk of false-positive results.

## 5. Conclusions

In this study, selexipag demonstrated a significant improvement in 6MWD, a significant reduction in oxygen requirements and eRVSP in patients with CTD-PAH and concomitant ILD. Importantly, treatment with selexipag was associated with a reduction in supplemental oxygen supplementation and no evidence of worsening gas exchange. These observations suggest that selexipag may confer a beneficial therapeutic option for CTD-PAH patients with concomitant ILD. Based on the provocative findings, further investigation in larger randomized controlled trials seems warranted.

## Figures and Tables

**Table 1:** Patient baseline characteristics.

Patient	1	2	3	4	5	6	7	8
Age	55	62	49	48	51	59	52	51
Gender	M	F	F	F	F	M	F	M
CTD type	MCTD	MCTD	Scleroderma	Scleroderma	Scleroderma	MCTD	Scleroderma	MCTD
CTD duration	2	4	3	2	4	1	5	2
PAH treatment	PDE-5-I + ERA	PDE-5-I + ERA	PDE-5-I + ERA	PDE-5-I + ERA	PDE-5-I + ERA	PDE-5-I + ERA	PDE-5-I + ERA	PDE-5-I + ERA
Cardiopulmonary hospitalizations	0	1	2	1	4	0	1	0
DLCO (%)	38	46	40	28	50	46	31	32
ILD								
Type	NSIP	NSIP	Fibrotic NSIP	Fibrotic NSIP	Fibrotic NSIP	Fibrotic NSIP	Fibrotic NSIP	Fibrotic NSIP
Treatment	Mycophenolate mofetil Hydroxychloroquine	Mycophenolate mofetil Hydroxychloroquine	Hydroxychloroquine	Hydroxychloroquine	Mycophenolate mofetil	Mycophenolate mofetil Prednisone	Hydroxychloroquine	Hydroxychloroquine

F: female; M: male; CTD: connective tissue disease; MCTD: mixed connective tissue disease; PAH: pulmonary arterial hypertension; PDE-5-I: phosphodiesterase type 5 inhibitor; ERA: endothelin receptor antagonist; DLCO: diffusing capacity of the lung for carbon monoxide, expressed as % predicted; ILD: interstitial lung disease; NSIP: nonspecific interstitial pneumonia.

**Table 2:** Right heart catheterization findings at the time of diagnosis.

Patient	1	2	3	4	5	6	7	8
RHC								
mPAP	45	33	41	52	35	39	50	29
PCWP	10	10	11	9	5	14	14	7
CO	5	3.9	5.2	3.2	4.1	2.9	2.9	5.3
PVR	7	5.9	6.5	13.4	7.3	12.4	12.4	4.2

RHC: right heart catheterization at the time of pulmonary hypertension diagnosis; mPAP: mean pulmonary arterial pressure (mmHg); CO: cardiac output (L/min); PVR: peripheral vascular resistance.

**Table 3:** Changes in 6MWD, NT-proBNP, RVSP, supplemental oxygen therapy, and FVC from baseline to 16 weeks after selexipag use.

Patient	1	2	3	4	5	6	7	8
6MWD								
Baseline	287	390	332	150	340	380	166	400
16 weeks	350	500	503	299	392	452	288	475
NT-proBNP								
Baseline	1100	743	1039	4203	922	522	6922	320
16 weeks	532	402	829	937	642	482	1825	187
eRSVP								
Baseline	77	60	71	90	55	65	88	60
16 weeks	58	43	44	67	44	47	52	41
O_2_ therapy								
Baseline	2 L	RA	2 L	4 L	2 L	RA	4 L	2 L
16 weeks	RA	RA	RA	2 L	2 L	RA	4 L	RA
Absolute FVC								
Baseline	1.8	2.1	2.3	1.7	1.9	1.6	2.6	2.1
16 weeks	1.8	1.9	2.3	1.8	1.5	1.6	2	2.2
Predicted FVC								
Baseline	63	59	67	49	62	54	68	62
16 weeks	61	54	63	47	65	62	62	57
Predicted FEV_1_								
Baseline	65	60	65	50	60	58	65	61
16 weeks	65	61	64	51	59	60	65	63
Absolute FEV_1_								
Baseline	1.9	2.2	2.1	1.8	1.8	1.8	2.4	2.0
16 weeks	1.9	2.3	2.2	1.9	1.7	1.9	2.4	2.2

6MWD: six-minute walking distance (meters); NT-proBNP: N-terminal prohormone of brain natriuretic peptide (pg/mL); eRVSP: estimated right ventricular systolic pressure (mmHg); RA: room air; FVC: forced vital capacity, absolute (liters) and predicted (%); FEV_1_: forced expiratory volume in one eecond, absolute (liters) and predicted (%).

**Table 4:** Summary of paired *t*-test results for clinical parameters post-selexipag treatment.

	Baseline Average	Average After 16 Weeks of Therapy	Difference	T-Score	*p*-Value
6MWD	305.62	407.37	101.75	−6.66	0.00029
NT-proBNP	1971.37	729.5	−1241.87	1.86	0.1048
eRSVP	70.75	49.5	−21.25	7.98	0.000093
Supplemental O_2_	2	1	1	2.65	0.033
Absolute FVC	2.01	1.89	−0.12	1.39	0.208
Predicted FVC	60.5	58.87	−1.62	0.96	0.371
Absolute FEV_1_	2	2.06	+0.06	−1.93	0.095
Predicted FEV_1_	60.5	61	+0.5	1.17	0.280

6MWD: six-minute walking distance (meters); NT-proBNP: N-terminal prohormone of brain natriuretic peptide (pg/mL); eRSVP: estimated right ventricular systolic pressure (mmHg); FVC: forced vital capacity, absolute in liter, and predicted in percentage; FEV_1_: forced expiratory volume in one second, absolute (liters) and predicted (%).

## Data Availability

The original contributions presented in the study are included in the article, further inquiries can be directed to the corresponding author.

## Abbreviations

The following abbreviations are used in this manuscript:

CTD-PAH	connective tissue disease-associated pulmonary arterial hypertension
ILD	interstitial lung disease
PH	pulmonary hypertension
CTD	connective tissue disease
RHC	right heart catheterization
6MWD	six-minute walk distance
eRVSP	estimated right ventricular systolic pressure
NT-proBNP	N-terminal pro b-type natriuretic peptide
PFTs	pulmonary function tests
PH-ILD	pulmonary hypertension associated with interstitial lung disease
WHO	World Health Organization
PAH	pulmonary arterial hypertension
PH-LHD	pulmonary hypertension due to left heart disease
PH-LD	pulmonary hypertension due lung disease
CTEPH	chronic thromboembolic pulmonary hypertension
IPF	idiopathic pulmonary fibrosis
HP	hypersensitivity pneumonitis
NSIP	nonspecific interstitial pneumonia
mPAP	mean pulmonary artery pressure
PCWP	pulmonary capillary wedge pressure
WU	wood units
DLCO	diffusing capacity of the lungs for carbon monoxide
FEV_1_	forced expiratory volume in one second
FVC	forced vital capacity
FDA	food and drug administration
MCTD	mixed connective tissue disease
PDE-5-I	phosphodiesterase-5 inhibitor
ERA	endothelin receptor antagonist
PVR	pulmonary vascular resistance
